# Magnetic propelled hydrogel microrobots for actively enhancing the efficiency of lycorine hydrochloride to suppress colorectal cancer

**DOI:** 10.3389/fbioe.2024.1361617

**Published:** 2024-02-21

**Authors:** Fengqi Jiang, Qiuyan Zheng, Qingsong Zhao, Zijuan Qi, Di Wu, Wenzhong Li, Xiaoke Wu, Conghui Han

**Affiliations:** ^1^ Department of Urology, Xuzhou Clinical School of Xuzhou Medical University, Xuzhou Central Hospital, Xuzhou, Jiangsu, China; ^2^ Department of General Surgery, Heilongjiang Provincial Hospital, Harbin, China; ^3^ School of Life Sciences, Jiangsu Normal University, Xuzhou, Jiangsu, China; ^4^ Department of Pharmacy, The Second Affiliated Hospital of Harbin Medical University, Harbin, China; ^5^ Postdoctoral Programme of Meteria Medica Institute of Harbin University of Commerce, Harbin, China; ^6^ Department of Pathology, Heilongjiang Provincial Hospital, Harbin, China; ^7^ Department of Obstetrics and Gynecology, First Affiliated Hospital, Heilongjiang University of Chinese Medicine, Harbin, China

**Keywords:** colorectal cancer, magnetic-driven, microrobots, lycorine hydrochloride, Fe_3_O_4_ particle

## Abstract

Research and development in the field of micro/nano-robots have made significant progress in the past, especially in the field of clinical medicine, where further research may lead to many revolutionary achievements. Through the research and experiment of microrobots, a controllable drug delivery system will be realized, which will solve many problems in drug treatment. In this work, we design and study the ability of magnetic-driven hydrogel microrobots to carry Lycorine hydrochloride (LH) to inhibit colorectal cancer (CRC) cells. We have successfully designed a magnetic field driven, biocompatible drug carrying hydrogel microsphere robot with Fe_3_O_4_ particles inside, which can achieve magnetic field response, and confirmed that it can transport drug through fluorescence microscope. We have successfully demonstrated the motion mode of hydrogel microrobots driven by a rotating external magnetic field. This driving method allows the microrobots to move in a precise and controllable manner, providing tremendous potential for their use in various applications. Finally, we selected drug LH and loaded it into the hydrogel microrobot for a series of experiments. LH significantly inhibited CRC cells proliferation in a dose- and time-dependent manner. LH inhibited the proliferation, mobility of CRC cells and induced apoptosis. This delivery system can significantly improve the therapeutic effect of drugs on tumors.

## 1 Introduction

Colorectal cancer (CRC) has always been a public health problem due to its high incidence rate and mortality. The data in the GLOBOCAN 2020 global report shows that CRC ranks third in terms of new cases (193,159,0 10%), and second in terms of cancer-related deaths (935,173 cases, 9.4%) (Ionescu et al., 2023). Currently, the treatment of patients with metastatic colorectal cancer (mCRC) mainly focuses on chemotherapy, supplemented by surgery and radiotherapy. With the development of therapeutic approaches, the survival period and quality of life of patients have been significantly improved (Sunakawa et al., 2016). However, overall, the 5-year survival rate of mCRC patients with mCRC remains low, at approximately 15% (Ohishi et al., 2023). Genetic mutations often lead to increased drug resistance in tumor cells, making existing treatment methods ineffective in controlling tumor growth and spread (Qunaj et al., 2023). Therefore, researchers have been searching for new treatment strategies to overcome this challenge (Cruz-Martins, 2023).

Compounds from plant sources have the advantages of low cost, high stability, high safety, and multi-targeting, which make them highly valuable in clinical applications (Avila-Carrasco et al., 2019). Lycorine hydrochloride (LH), as an active alkaloid, is extracted from the medicinal plant Lycoris radiate (Shi et al., 2021). In recent years, the research on the anti-tumor effect and mechanism of LH has been increasing. It has been found that LH can exert anti-tumor activity through multiple pathways and multiple signaling pathways, which is specifically reflected in the regulation of the occurrence and development of multiple tumors through one pathway, or the regulation of the same tumor through different signaling pathways (Ji et at., 2017; Li et al., 2020; Li et al., 2021). Although there have been many reports on the anti-tumor effects of LH, there have been relatively few studies on its role in CRC.

When normal cells are affected by synthetic lethal drugs and lead to serious side effects, synthetic lethality shows its limitations and shortcomings. As a result, patients may be forced to interrupt treatment due to strong side effects during treatment (Chen et al., 2020). In contrast, the precision drug delivery system may enhance the synthesis of lethal drugs in the tumor site while reducing side effects (Li et al., 2023). Micro/nano-robots have been innovatively adopted to solve problems related to Brownian motion and viscous forces, and to utilize different power sources for movement (Chen et al., 2018; Wang et al., 2021; Zhao et al., 2022). The magnetic-driven microrobots that achieves precise manipulation by changing the strength and direction of the magnetic field has great potential for clinical application in drug treatment of tumors, which has aroused great interest among researchers (Li et al., 2017; Wang et al., 2020; Ji et al., 2021; Zhang et al., 2023; Li et al., 2023). By precisely controlling the magnetic field, microrobots can be guided to the tumor site and release drugs, which not only greatly improves the therapeutic effect of drugs, but also significantly reduces a series of problems such as drug cytotoxicity and serious complications (Chen et al., 2018; Mu et al., 2022; Wang et al., 2022). This technology is expected to provide new solutions for tumor treatment in the future (Wang et al., 2021).

## 2 Materials and methods

### 2.1 Preparation of microfluidic chip

The microfluidic chip device is consisted of PDMS construction, capillary tubes, dispensing needles, and rubber tubes. The rubber tubes are used as microchannels for the dispersed phase and continuous phase. One end of the capillary tube is inserted into the PDMS body, and the other end is connected to the rubber tubes. In experiment, the fluid flows into the PDMS construction through the rubber tubes and capillary tube in sequence, where the micro droplets are sheared, and then flows out collectively through the rubber tube at the other end. All openings on the device surface are sealed with leak-proof oil sealant and left to stand for 15 min for the sealant to cure. The device is then stored in a cool and dry place.

### 2.2 Preparation of continuous phase and dispersed phase

#### 2.2.1 Continuous phase (oil phase):

20 μL of Tween 20 (surfactant) was dropped into 20 mL of vegetable oil and stir to mix thoroughly. Then the mixed liquid was placed in a vacuum drying box, evacuate to 1,000 Pa for 30 min to remove bubbles in the mixed solution to obtain the continuous phase fluid required for the experiment.

#### 2.2.2 Dispersed phase (water phase):

(1) 300 mg of gelatin (glue strength: ∼250 g Bloom, Aladdin, China) was added into 7 mL of deionized (DI) water and then was stirred for 30 min at 40°C using magnetic stirrer until the gelatin was dissolved.
(2) 20 mg of photoinitiator (2-hydroxy-4′-(2-hydroxyethoxy)-2-methylpropiophenone) (Macklin, Shanghai, China) was added into 3 mL of DI water. Then mixed liquid was stirred for 10 min at 30°C using magnetic stirrer to obtain photoinitiator aqueous solution.
(3) The gelatin aqueous solution and the photoinitiator aqueous solution were mixed, and 0.1 mL of acid-base buffer was added.
(4) The Fe_3_O_4_ particles (20 nm) and LH were added into the mixed solution, and adjust the concentrations to 2.5% and 20 μM, respectively.

### 2.3 External magnetic field device

A three-degree-of-freedom Helmholtz coil, a multi-function data acquisition device, and three single-channel output power amplifiers jointly constitute the external rotating uniform magnetic field. By controlling the current and voltage of the Helmholtz coil, a circular external rotating uniform magnetic field can be generated on any plane in three-dimensional space to drive the microrobots to move in different ways (Wang et al., 2023). In the experiment, the cell-culture dish was placed in the center of the three-dimensional Helmholtz coil, where the magnetic field intensity was evenly distributed. After adding the drug-loaded microrobot, turning on the magnetic field can drive the microrobot to move towards the cells.

### 2.4 Cells and reagents

CRC cell lines DLD-1 and LoVo, as well as human intestinal epithelial cell line NCM460, are provided by Fuheng Cell Centre (Shanghai, China). LoVo cells were cultured in RPMI 1640 medium (Gibco; Thermo Fisher Scientific, Inc.), while DLD-1 and NCM460 cells were cultured in Dulbecco’s modified Eagle’s medium (DMEM; Thermo Fisher Scientific, Inc., Waltham, MA, United States). Both media were supplemented with 10% fetal bovine serum (FBS; Gibco; Thermo Fisher Scientific, Inc.) and 1% antibiotic (Sigma-Aldrich, United States). All cells were cultured in a standard humidified incubator at 37°C under an atmosphere with 5% CO_2_. LH (cat. no. L101559) was purchased from Aladdin Industrial Corporation (Shanghai, China) and was dissolved in dimethyl sulfoxide (DMSO) to prepare a stock solution of 77.16umol/L.

### 2.5 MTT assay

The cytotoxicity of LH was conducted by MTT assay. Cells were incubated in 96-well plate (5×10^3^ per/well) for 24 h, then treated with different concentrations of LH (0–100 μM) for 48 h. After staining with MTT solution (5 mg/mL, 20 μL/well, 4 h) (Sigma-Aldrich, Merck KGaA, Darmstadt, Germany), 150 μL of DMSO was added to each well to solubilize the formazan crystals. A microplate reader (ELx808, BioTek Instruments, Winooski, VT, United States) was employed to detect the plate at the wavelength of 490 nm. Three biological experiments were performed.

### 2.6 Plate colony-forming assay

CRC cells (0.5 × 10^3^) were cultured in 6-well plates for 24 h. Three groups were set up, including a negative control group (NC), LH-alone (20 μM), and a magnetic microrobot drug-loading group (LH-robot) (20 μM). Treatments were conducted on days 2, 4. After 10 days, the cells were fixed with methanol and stained with a 0.5% crystal violet solution. Three biological experiments were performed.

### 2.7 Wound healing assay

DLD-1 and LoVo cells (90% confluence) were scratched with a sterile 200-µL pipette tip and then treated with LH and LH-robots (20 μM) separately. After 48 h, the width of the scratch was observed. The scratch was imaged under a microscope (magnification, ×100). The widths of the scratches were analysed with ImageJ V1.8.0 (NIH, Bethesda, MD, United States). Three biological experiments were performed.

### 2.8 Transwell assay

The invasion experiment was conducted using a Transwell chamber. Before cells seeding, 50 μL of Matrigel was added to the upper chamber to coat the polycarbonate membrane. DLD-1 and LoVo cells were treated with LH alone and LH-robots (20 μM) separately and then cultured for 24 h. After resuspension in FBS-free medium, they were inoculated into the upper chamber (1×10^4^ cells/200 µL medium per well). Then, the lower chamber containing 700 µL of medium with 10% FBS was used as a hemoattractant. After 48 h of incubation, cells on the polycarbonate membrane were wiped off, and then methanol was used to fix the cells penetrating to the dorsal side, followed by staining with a 0.5% crystal violet solution. Under a microscope (magnification, ×100), 5 randomly selected fields were quantitatively analysed. Three biological experiments were performed.

### 2.9 Apoptosis assay

DLD-1 and LoVo cells were seeded in 6-well plates (2.5×10^5^ per/well) and allowed to adhere overnight. Then, LH alone and LH-robots (20 μM) were added separately, and the cells were incubated for 48 h before being harvested and stained with an Annexin V-FITC/PI Apoptosis Detection kit (catalog no. FXP018; 4A Biotech). FACS DiVa 6.1.3 (BD Biosciences, Franklin Lakes, NJ, United States) was used to analyze apoptosis. Three biological experiments were performed.

### 2.10 Statistical analysis

All data were shown as means ± SD via at least triplicate samples. A two-tailed, Student’s t test was used for testing the significance between two groups. Statistical analyses were performed using GraphPad PRISM 9 (GraphPad Software, Inc.) A one-way analysis of variance (ANOVA) with Dunnett’s test was performed to test the significance for multiple comparisons. A statistical significance was assumed at *p* < 0.05.

## 3 Results

### 3.1 Preparation of magnetic drive microrobot delivery system

**FIGURE 1:**
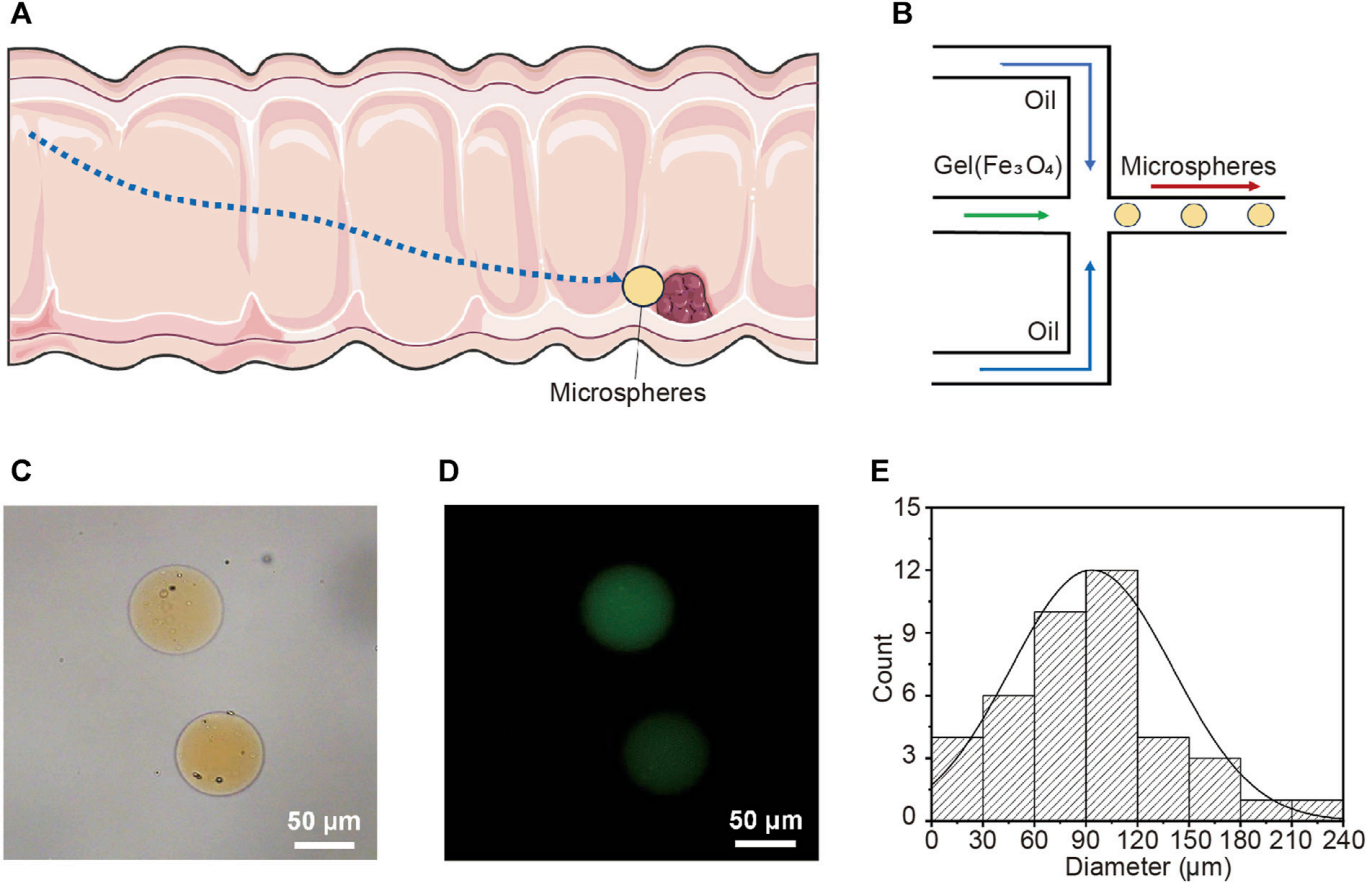
Targeted drug release in intestine using magnetic-driven hydrogel microrobots. **(A)** Schematic overview of magnetic-driven hydrogel microrobots for drug release in intestine. **(B)** The fabrication of magnetic-driven hydrogel microrobots using microfluidic chip. **(C)** The optical microscopy image of magnetic-driven hydrogel microrobots. **(D)** Fluorescence microscopic images of magnetic-driven hydrogel microrobots. **(E)** Particle size distribution of magnetic-driven hydrogel microrobots.

Autonomous micro/nano-robots can propel and navigate in various liquid media, and are expected to provide revolutionary technological advancements for drug delivery, microsurgery, and micro/nano-engineering (Xiao et al., 2022; Yang et al., 2022; Wang et al., 2021; Gao et al., 2021; Zhang et al., 2023). Magnetic nanorobots show great potential in practical biomedical applications due to their wireless fuel-free actuation, strong propulsion, precise motion control, and high biocompatibility (Wang and Zhang, 2021; Xie et al., 2020; Zhou et al., 2021; Yang et al., 2023). This study describes the preparation of magnetic-driven hydrogel microrobots. It has the capability to transport drug LH through the digestive channel and into the intestine. Subsequently, the microrobot, driven by an external magnetic field, precisely maneuvers to the target lesion location for drug release and treatment (Figure 1A). This application demands that the microrobot is capable of loading drugs, exhibiting magnetic responsiveness, and possessing a biocompatible structure. Additionally, the microrobot should have a disintegrable body structure for drug release. Figure 1B illustrates the fabrication process of the magnetic-driven hydrogel microrobot. The hydrogel microrobot is fabricated using a microfluidic chip based on the principle of flow focusing. In the flow convergence device, the dispersed phase fluid and continuous phase fluid pass through a narrow region under pressure. At the micro-droplet generation site, there are three fluid streams, with the continuous phase fluid symmetrically distributed on both sides. The dispersed phase fluid in the middle is focused and sheared by the continuous phase fluids on both sides, forming micro-droplets. The micro-droplets are subsequently cured and solidified on the surface under UV light exposure, thus transforming into magnetic-driven hydrogel microrobots. In this process, the continuous phase is plant oil, and the dispersed phase is a gelatin aqueous solution containing photoinitiator, along with Fe_3_O_4_ particles and the LH.

The optical microscopy image shown in Figure 1C displays the spherical geometry of the microrobots. The clearly visible yellow microparticles inside the microrobot indicate the successful loading of Fe_3_O_4_ particles. The fluorescent microscope image (Figure 1D) illustrates that the desired drug loading has been achieved. The loading of drugs and Fe_3_O_4_ particles decreased in the homogeneity of the gelatin solution as the dispersed phase, leading to a lack of strict uniformity in the size of the generated microrobots. Figure 1E presents the statistical analysis of the size distribution of the prepared microrobots, indicating that the size distribution follows a generally normal distribution. The average diameter is approximately 90 μm, predominantly distributed within the range of 30∼120 μm, exhibiting a high degree of monodispersity. In the microfluidic chip, the generation rate and size of microrobots can be modulated by altering the flow rate ratio, viscosity, and channel dimensions of the continuous and dispersed phase fluids.

**FIGURE 2:**
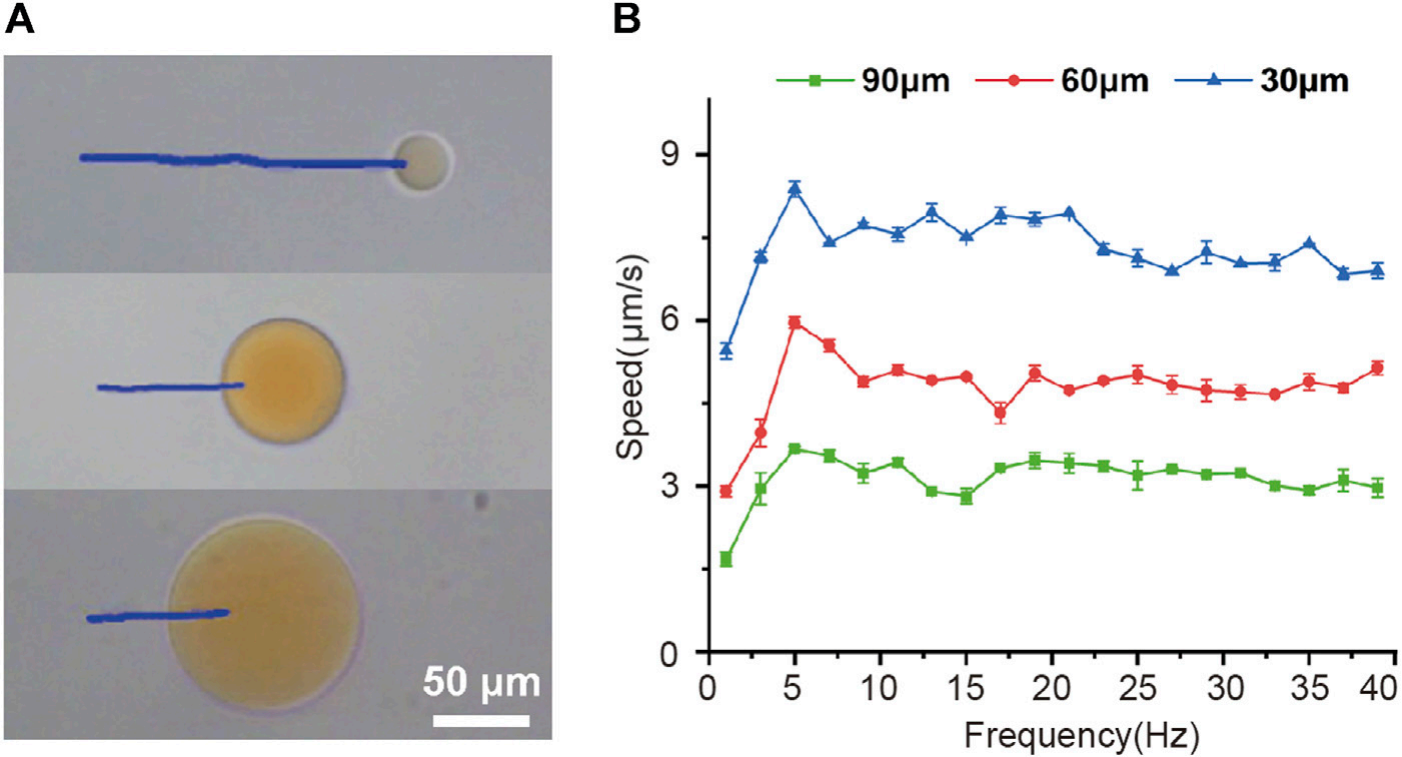
Magnetically actuated motility of magnetic-driven hydrogel microrobots. **(A)**Tracking lines illustrating the traveled distances of microrobots of different sizes over a 14 s period in a rotating uniform magnetic field of 15 mT and 5 Hz. **(B)** The velocity of microrobots of different sizes varied with the drive frequency from 2 to 40 Hz.

Then the effect of frequency of the rotating magnetic field on the velocity of magnetic-driven hydrogel microrobot was investigated experimentally as well. Figure 2A shows the trajectories of different-sized microrobots over a period of 14 s under the conditions of a magnetic force of 15 mT and a driving frequency of 5 Hz. It can be seen that larger microrobots exhibit smaller movement speeds. To further reveal the magnetic field-driven motion performance of microrobots, we explore the variation of microrobots’ velocity with the magnetic frequency increased from 2 to 40 Hz and shown in Figure 2B, for a 30 μm microrobots, the speed increased linearly with the driving frequency and reached a maximum velocity of 8.4 μm/s at 5 Hz, further increasing the frequency reduced the velocity. Such a maximum synchronized frequency is called step-out frequency (Xie et al., 2019; Yu et al., 2019). The occurrence of the out-of-step phenomenon and the increase in drag caused by the increasing speed are the reasons we speculate for this variation. After the step-out phenomenon occurred, the speed of the microrobot fluctuates within a certain range as the frequency of the magnetic frequency increase. Furthermore, the 60 and 90 μm microrobots showed the same movement performance and obtained the highest velocities of 6 μm/s and 3.7 μm/s, respectively. This magnetically actuated motility of magnetic-driven hydrogel microrobots of different sizes can provide guidance for the customization of microrobots for different working conditions.

**FIGURE 3:**
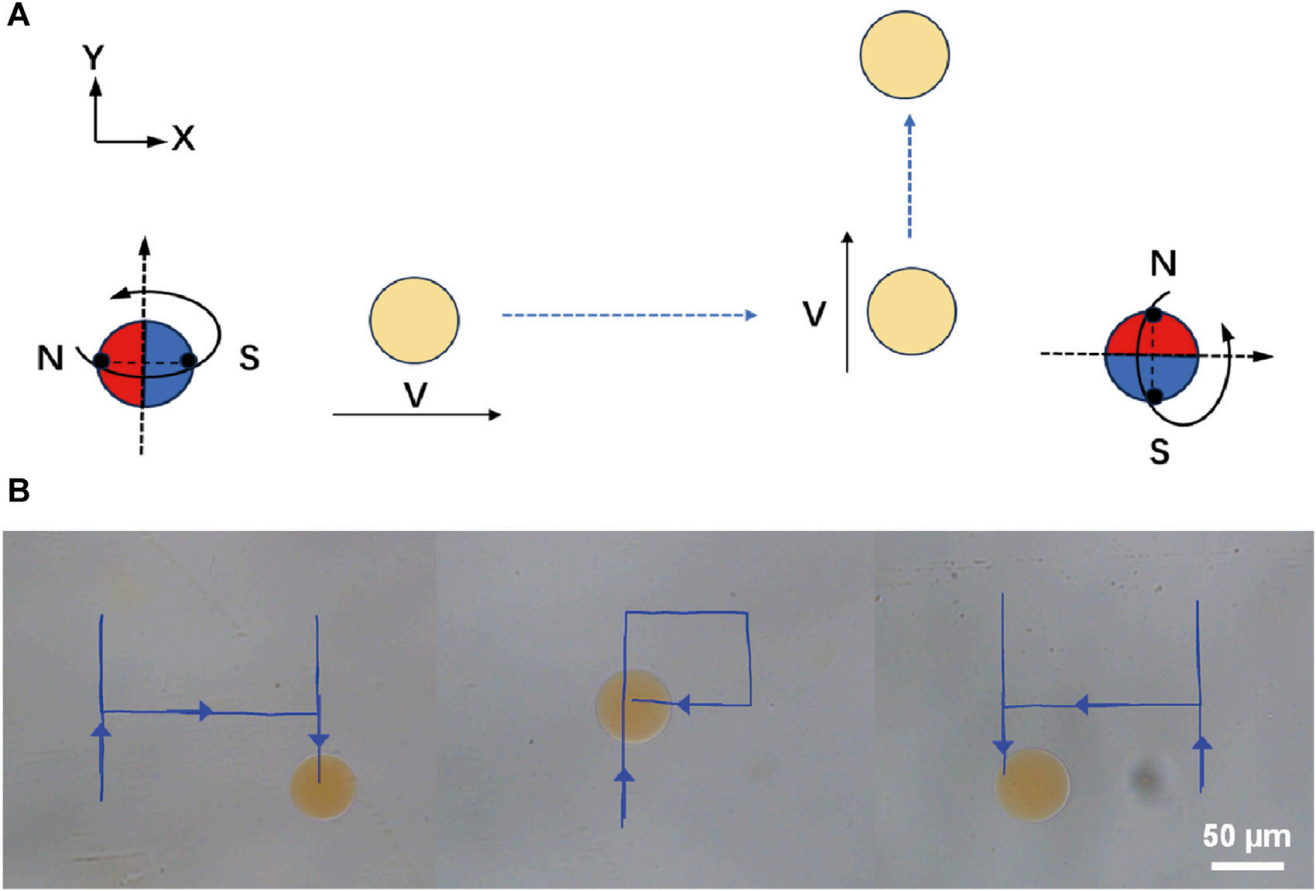
Controllable and flexible motility performance of magnetic-driven hydrogel microrobots. **(A)** Change of the direction of movement of the microrobots caused by changing the magnetic field. **(B)** Controllable motion of microdimer swimmer.

For the application of micro/nano-scale robots in precision medical procedures, the ability of remote driving has very attractive characteristics (Chirarattananon et al., 2014; Xie et al., 2019; Aram et al., 2022; Zhang et al., 2022). Here, we demonstrate the remote locomotion of Janus magnetic-driven hydrogel microrobots. Figure 3A illustrates the control strategy of three-dimensional rotating magnetic field generated by the three degrees of freedom Helmholtz coil and corresponding movement of microrobots. First, a circularly polarized rotating magnetic field applied in the X-Z plane excited the microrobot rolled along X-axis. When the rotating magnetic field was changed and applied in the Y-Z plane, the locomotion direction of microrobot changed to the Y-axis. The propulsion direction of the microrobot could be altered by changing the direction of rotating magnetic field manually. Based on this rule, we realized the controllable trajectory movement of microrobot by modulating the magnetic field. As shown in Figure 3B, the microrobot walked along letter “H”-, “P”-, and “H”-shaped trajectory. The microrobot realized flexible direction switching under the drive of magnetic field of 15 mT and 5 Hz. This remote controllable movement capability provides support for targeted locomotion of magnetic-driven hydrogel microrobots on the complex surface of the intestine.

### 3.2 Magnetic-driven microrobots significantly enhances the ability of LH to inhibit CRC cells

**FIGURE 4:**
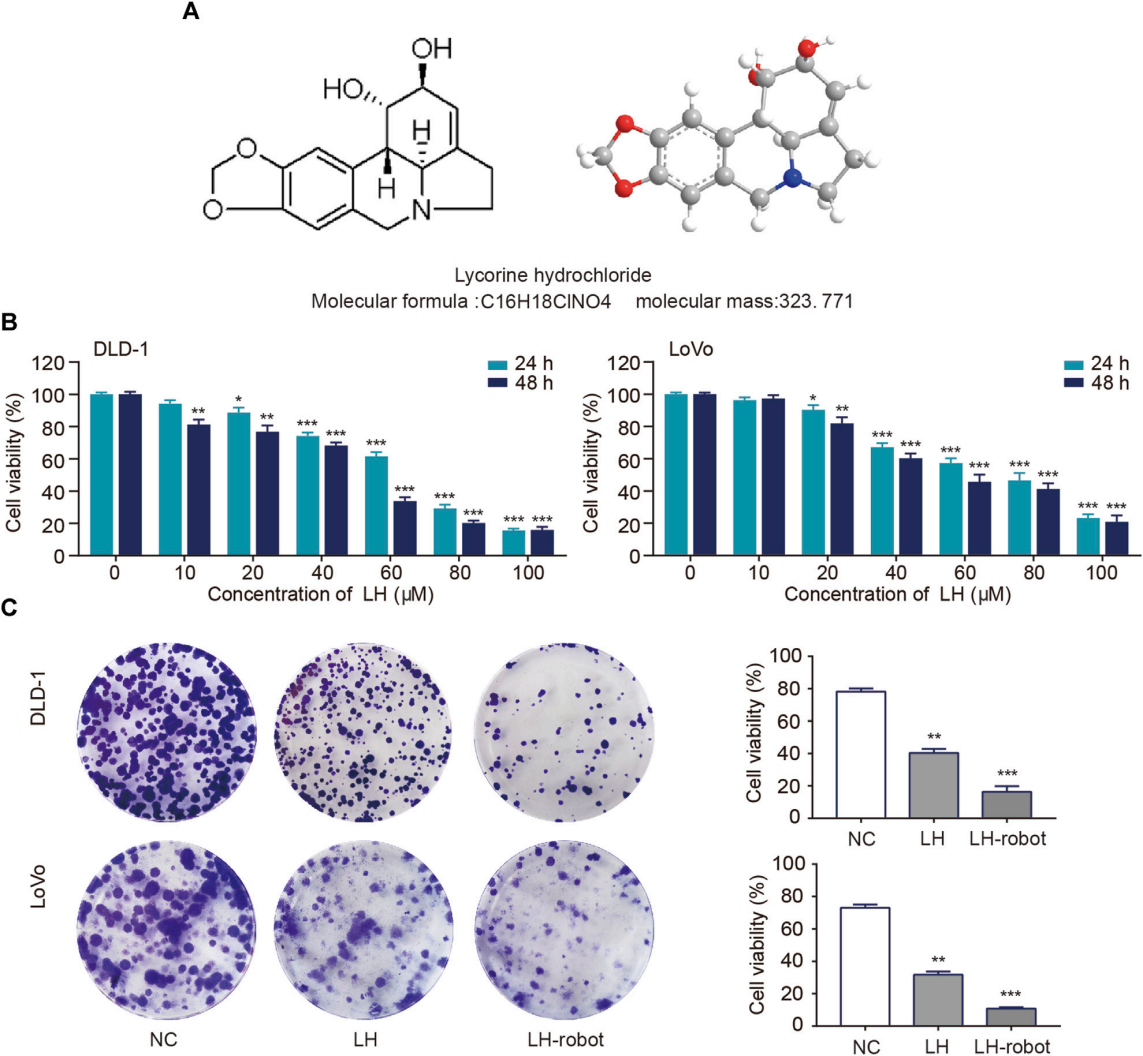
Effect of LH and LH-robot on CRC cell proliferation. **(A)** Chemical and three-dimensional structures of LH. **(B)** An MTT assay was performed to measure CRC cell viability following LH (0–100 µM) treatment. **(C)** The influence of LH alone and LH-robot on the colony formation of CRC cells. *p < 0.05 and **p < 0.01 and ***p < 0.001 vs. NC group.

• The chemical structure formula and 3D structure of LH are shown in respectively. Cell proliferation was assessed using MTT and colony formation assays (Chan et al., 2023). CRC cells were exposed to multiple concentrations (0–100 µM) of LH for 24 and 48 h. MTT assay results showed that LH significantly inhibited CRC cells proliferation in a dose and time-dependent manner (Figure 4B). Notably, LH showed minimal cytotoxicity towards NCM460 cells (Supplementary Figure S1). We chose a non-lethal concentration of 20 µM of LH and compared the effects of single drug administration with microrobot-based drug delivery on CRC cells. A large number of previous studies (Zhang et al., 2021; Zhou et al., 2021) have shown that Fe_3_O_4_ as a component of magnetic drive micro-nano robot will not cause biological tissue and cell damage, so the blank control group in this study containing a small dose of Fe_3_O_4_ will not cause additional effects. The colony formation experiment showed that the drug under the control system of magnetic microrobots had a more significant ability to inhibit the proliferation of CRC cells (Figure 4C).

**FIGURE 5:**
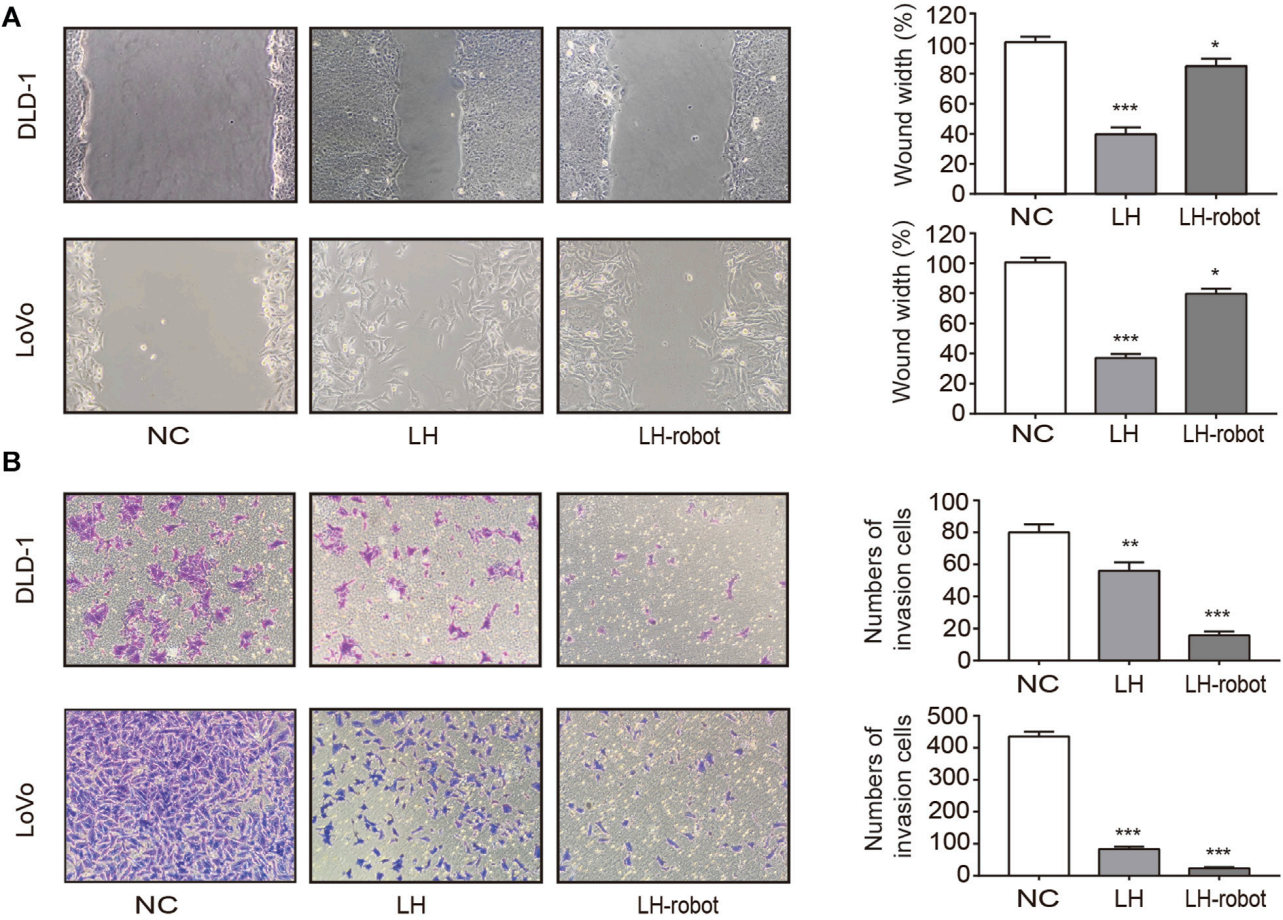
Effect of LH and LH-robot on CRC cell mobility. **(A)** Wound healing assay of LH alone and LH-robot on CRC cells at non-toxic concentrations (20µM, 24 h). Magnification, ×100. **(B)** Invasion assays of CRC cells pretreated with LH and LH-robot at non-toxic concentrations (20µM, 24 h). Magnification, ×100. *p < 0.05 and **p < 0.01 and ***p < 0.001 vs. NC group.

Metastasis is the process by which cancer cells grow in organs far away from their primary organ, and is the deadliest manifestation of cancer (Xia et al., 2023). The vast majority of cancer patients die from metastatic disease, rather than primary tumors (Chen et al., 2019). Tumour cell mobility is essential for metastasis and is typically assessed by wound healing and Transwell assays (Jin et al., 2019). As shown in Figure 5A the wound healing speed in the cells treated with LH-robot was significantly slower. The Transwell assay results showed a significant decrease in the invasion ability of the experimental group cells, especially in the magnetic microrobot-based drug delivery group (Figure 5B).

**FIGURE 6:**
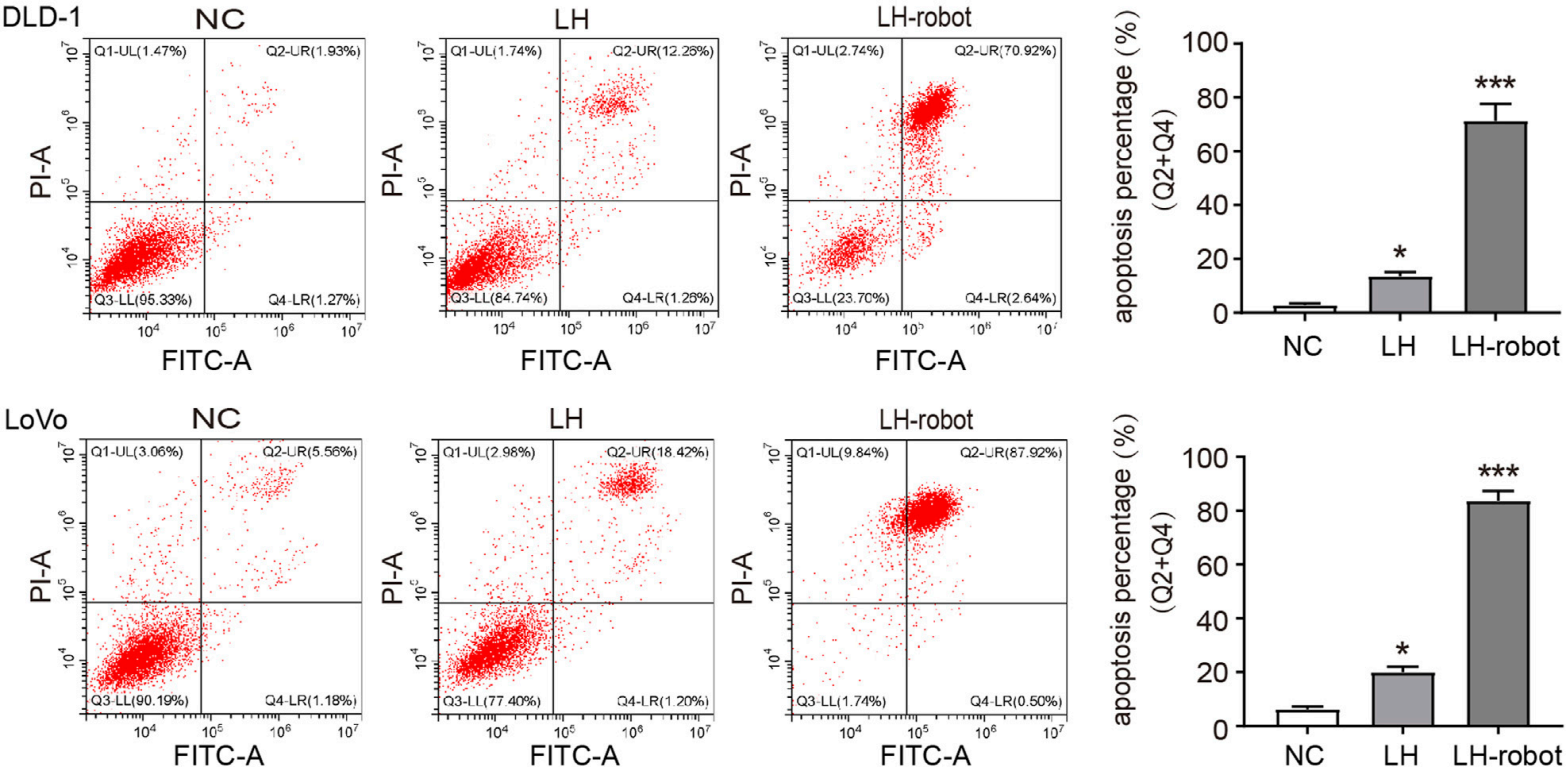
The apoptosis of CRC cells after treatment of LH and LH-robot separately. *p < 0.01 and ***p < 0.001 vs. NC group.

Apoptosis is a programmed cell death that balances the ratio of cell survival and death. Cancer cells have the ability to escape apoptosis, so aimed at inducing cancer apoptosis is a very important direction for treating cancers (Elbanna et al., 2021). Cytotoxic chemotherapy and radiotherapy attempt to trigger apoptosis through endogenous pathways by acting on cell division and/or directly damaging DNA, and are currently the main methods for treating cancer through the induction of apoptosis mechanisms (Wanner et al., 2020; Barroso et al., 2023). In our experiments, we observed that LH has the ability to induce apoptosis in CRC cells, and the magnetic propelled hydrogel microrobots significantly enhance this ability (Figure 6).

## 4 Conclusion

We have fabricated a magnetic driven, biocompatible hydrogel microrobot loaded with Fe_3_O_4_ particles. Through the regulation of external magnetic fields, the movement of microrobots can be precisely controlled, enhancing the anticancer ability of LH on CRC cells. This technology can achieve precise delivery and efficient utilization of drugs, thereby reducing the toxic side effects and improving the therapeutic effect. Therefore, microrobot technology has broad prospects in cancer treatment.

## Data Availability

The raw data supporting the conclusion of this article will be made available by the authors, without undue reservation.
