# Supplementary figures and images for: Magnetic propelled hydrogel microrobots for actively enhancing the efficiency of lycorine hydrochloride to suppress colorectal cancer
Source: Front Bioeng Biotechnol. 2024 Feb 21;12:1361617. doi: 10.3389/fbioe.2024.1361617 (PMC10915283; doi:10.3389/fbioe.2024.1361617)

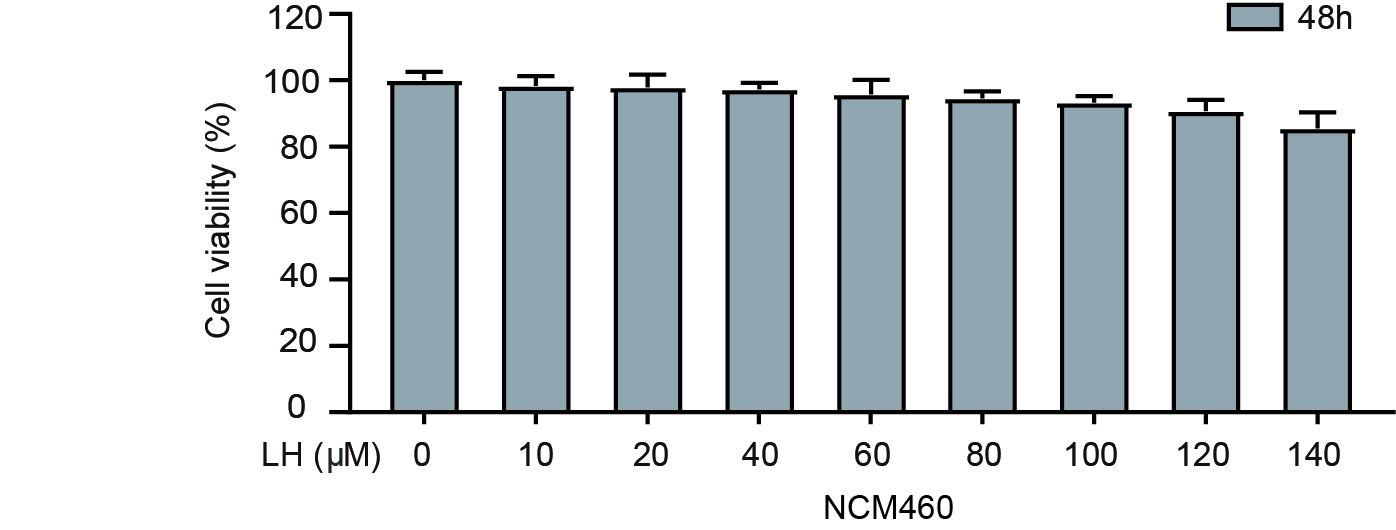

Supplement: Supplementary file 1 [file Image1.JPEG]
